# Caregiving Barriers In Parkinson’s Disease With Cognitive Impairment: A Qualitative Study

**DOI:** 10.1093/geroni/igaf122.2276

**Published:** 2025-12-31

**Authors:** Yuncheng Huang, Yijing Hua, Jing Wang, Bei Wu, Juan Li

**Affiliations:** Huashan Hospital Fudan University, Shanghai, China (People’s Republic); Huashan Hospital Fudan University, Shanghai, China (People’s Republic); University of New Hampshire, Durham, New Hampshire, United States; NYU Shanghai, Shanghai, China (People’s Republic); Huashan Hospital Fudan University, Shanghai, China (People’s Republic)

## Abstract

**Background:**

Parkinson’s disease (PD) is often accompanied by cognitive impairments (CI), which lead to a decline in the quality of life for patients and increasing pressure on caregivers. However, there are currently few qualitative studies exploring the care barriers for patients with Parkinson’s disease and cognitive impairment (PD-CI).

**Objective:**

This study aimed to identify unmet care needs, analyze caregiving challenges, and propose solutions by triangulating perspectives from people with Parkinson’s, caregivers, and healthcare professionals.

**Methods:**

Semi-structured interviews were conducted with ten people with Parkinson’s and cognitive impairment, ten family caregivers, and eleven healthcare professionals in the Huashan Hospital, Fudan University in Shanghai, (2024.10-2025.1). Participants were recruited through purposive sampling, with cognitive impairment identified by healthcare professionals. Interviews were audio-recorded, transcribed, and analyzed using reflexive thematic analysis following Bronfenbrenner’s ecological systems theory.

**Results:**

The study extracted four themes: ①Microsystem - diagnostic uncertainty, stigma and self-identity, self-management challenges, and motivation difficulties; ② Mesosystem - caregivers’ struggles (communication barriers, household dynamics, caregiving stress, and difficulties in care coordination); ③ Exosystem - Social challenges; ④ Macrosystem - the influence of cultural and structural factors (care access and resource distribution).

**Conclusion:**

The care pressure of patients with PD-CI is great, and they face many challenges. It is possible to try to build a “patient-medical staff-family member” co-participatory care model to improve the quality of care and quality of life of patients with PD-CI.

